# Physical Layer Security in Multimode Fiber Optical Networks

**DOI:** 10.1038/s41598-020-59625-9

**Published:** 2020-02-17

**Authors:** Stefan Rothe, Nektarios Koukourakis, Hannes Radner, Andrew Lonnstrom, Eduard Jorswieck, Jürgen W. Czarske

**Affiliations:** 1 Technische Universität Dresden, Faculty of Electrical and Computer Engineering, Laboratory of Measurement and Sensor System Technique, 01062 Dresden, Germany; 2 Technische Universität Braunschweig, Faculty of Electrical Engineering, Information Technology, Physics, Institute for Communications Technology, 38106 Braunschweig, Germany

**Keywords:** Fibre optics and optical communications, Information theory and computation

## Abstract

The light propagation through a multimode fiber is used to increase information security during data transmission without the need for cryptographic approaches. The use of an inverse precoding method in a multimode fiber-optic communication network is based on mode-dependent losses on the physical layer. This leads to an asymmetry between legitimate (Bob) and illegitimate (Eve) recipients of messages, resulting in significant SNR advantage for Bob. In combination with dynamic mode channel changes, there are defined hurdles for Eve to reconstruct a sent message even in a worst-case scenario in which she knows the channel completely. This is the first time that physical layer security has been investigated in a fiber optical network based on measured transmission matrices. The results show that messages can be sent securely using traditional communication techniques. The technology introduced is a step towards the development of cyber physical systems with increased security.

## Introduction

The amount of exchanged data via internet has increased exponentially in recent years^1^. Following this trend, the amount of sensitive data has increased in the same way and thus the importance of information security. Today the most commonly used technique warranting secure communication is based on secret cryptographic keys, which exploit the complexity of multiplying large prime numbers^2^. However, cryptographic algorithms are facing several challenges. First, they are vulnerable against unexpected technological developments, as parallel networks of computers are breaking codes that have been considered safe in finite time using high-performance computers^3,4^. Second, the demands which are addressing the physical embodiments of cryptographic algorithms, e.g. one-way functions, go beyond the constraints of conventional semiconductor technology. Third, a thief stealing a digital key can go unnoticed.

Overcoming the drawbacks of computational cryptography, investigations of physical cryptographic methods have been made using physical parameters for secret key generation^5–8^. Physical unclonable functions (PUFs) are physical objects that cannot feasibly be copied due to their comprehensive number of degrees of freedom. Therefore, they have been studied as a physical one-time pad^9^ and as a secure physical authentication protocol^3,10–12^, respectively. In both scenarios optical PUFs were generated by illuminating scattering media.

One-time pads are information theoretically secure; however, the realization is highly impractical as both the sender (e.g. *Alice*) as well as the receiver (e.g. *Bob*) of a message cannot synchronize their channel without having exactly the same *unique* PUF.

In applications where PUFs were utilized to create secure authentication, the security relies on the difficulty of cloning the optical response^3^, as well as in combination with a low mean photon number^11,12^. This physical encryption technique always assumes that Alice and Bob have access to the PUF in an initial calibration step, which is not observed by a possible eavesdropper (e.g. Eve). This is a major problem, as due to statistical fluctuations in the light path caused by temperature, mechanical stress and local phase-shifts, the PUF is changing in time. Consequently, Alice and Bob need to recalibrate their channel, which is not practical when Eve is not supposed to watch the calibration procedure. This relationship represents a significant limitation of secret key-based schemes: there is always an interaction between transmitter and receiver needed. However, if there is a feedback channel available more sophisticated schemes are possible based on the problem of secret-key agreement via random sources, which was introduced in^13^ and followed by^14,15^.

Quantum Key Distribution (QKD) is a method intending to provide secure communication. It uses a cryptographic protocol that employs procedures of quantum mechanics. This approach gives two parties of a communication network the possibility to generate a secure key, which is only known to them. It is exchanged between them and is used to encrypt and decrypt messages. QKD utilizes the inimitability of unknown quantum states to make the reconstruction of encrypted messages impossible^16,17^. Even though QKD offers an unconditionally secure data transmission, serious problems arise when combining QKD signals with optical amplifier noise and classical communications^18,19^ on a conventional fiber optical infrastructure. Data transmission distances in quantum networks are mainly limited due to the difficulty of quantum repeater realization. The reason for this is that quantum states cannot be copied^20^ and a repeater in the classical sense of communications engineering is not feasible. However, it is possible to entangle a photon that is in one quantum state with another and to forward it^21^. With increasing number of such entanglements, it becomes more difficult to maintain the quantum state, as also with quantum computers^22^. This limits the applicability of quantum states in large scaled networks.

The use of multimode fibers (MMF) in fiber optical networks is regarded as a promising approach to increase data rates significantly, even in long-haul transmission systems^23^. However, the phenomenon of mode scrambling inside a MMF was considered as a hurdle for the MMF usage for a long time. Once coherent light is sent into the MMF on one side of the fiber, it will appear as a granulated structure on the other side of the MMF called speckle pattern. This barrier can be overcome with the development of wavefront shaping (WS)^24^. Firstly, it became feasible in optical engineering to control the propagation of light through fluctuating surfaces by WS^25^, secondly the light control through MMFs has not only become possible, but much more important. Nowadays, the MMF is a key device in several fields of research. They are used in biophotonical applications^26–33^ to gain access to hard-to-reach areas due to their flexibility and high number of degrees of freedom in a minimum space. These properties are particularly helpful for image transmission using the MMF as an ultrathin endoscope. In addition, achievable data rates in communication networks can be significantly increased using MMFs, since they can be used to develop novel multiplexing techniques in which space is a scalable parameter^34–37^.

Scattering of light in multimode fibers is often viewed as interference, but they can open the door to increase the level of information security as the scattering characteristics are random. Therefore, by controlling the transmission channel between Alice and Bob using WS, Eve, who is tapping off the signal somewhere in the center of the fiber, will only receive a scrambled speckle pattern. Additionally, this idea has been investigated in combination with a low photon number, so that Eve only receives a fraction of information^2^. However, this approach has two major drawbacks. On the one hand, a low-photon source is used, which is impractical with regard to desired transmission distances in fiber optical networks. On the other hand, it is assumed that Eve has no access to the transmission channel between Alice and Bob during the calibration step. However, in realistic scenarios, it is unknown whether an eavesdropper is able to witness the calibration or not.

In this paper, a novel approach to enhance the information security in fiber optical networks using physical layer security (PLS) is introduced, in which Eve is allowed to witness the calibration between Alice and Bob^38^. PLS is a technique in which information security is made possible not by the generation of a cryptographic key but by the physical properties of the transmission channels of the MMF itself^39^. As introduced in^18^, the modes supported by the MMF are unevenly leaving the MMF if someone is tapping off light between Alice and Bob. This phenomenon is called mode dependent loss (MDL)^40^ and is the key assumption of the introduced model. In the developed setup a conventional coherent light source is used. Since Eve is allowed to have access to the communication channel during the calibration phase between Alice and Bob, she has knowledge of the transmission matrix (T) between Alice and her, as well as the T between Alice and Bob. The scientific question to be answered in this paper is how to ensure secure communication between transmitter and receiver in a MMF optical network, even if an eavesdropper is present during the calibration phase between transmitter and receiver. For this purpose, measured Ts are used to investigate the possible advantages of MDL-based PLS in a simulation.

## Results

### Principle of MMF based PLS

In one calibration step, Alice measures the T of the MMF and thus all available channels. The complete light transmission characteristic between input ${\overrightarrow{\underline{x}}}_{A}$ on Alice’s side and output ${\overrightarrow{y}}_{B}$ on Bob’s side of the MMF is described by the MMF’s T:

1 $${\overrightarrow{y}}_{B}={T}_{AB}\cdot {\overrightarrow{\underline{x}}}_{A}+{n}_{B},$$

where T_AB_ is the T between Alice and Bob and ${n}_{B}$ is the measurement noise on Bob’s side. The T measurements are proceeded using the MMF mode domain as the basis of the T. Thus a MMF supporting N modes results in an N × N T and an 1 × N dimensioned input vector ${\overrightarrow{\underline{x}}}_{A}$. This means that the individual MMF modes are representing the available channels for Alice. The MMF under test supports 55 modes and has a step-index refractive index profile. The input vector ${\overrightarrow{\underline{x}}}_{A}$ is defined as follows to ensure an average constant transmission power:

2 $${\overrightarrow{\underline{x}}}_{A}=\frac{{\overrightarrow{x}}_{A}}{||{\overrightarrow{\underline{x}}}_{A}||}.$$

Alice is able to undo the scrambling property of the MMF by inverting the T. In the following, inverted matrices are marked with a superscript †. After superimposing the inverted T ${T}_{AB}^{\dagger }$ to the input signal ${\overrightarrow{\underline{x}}}_{A}$, the new input $\widehat{{\overrightarrow{\underline{x}}}_{A}}$can be described as:

3 $$\widehat{{\overrightarrow{\underline{x}}}_{A}}={T}_{AB}^{\dagger }\cdot {\overrightarrow{\underline{x}}}_{A}\,,$$

with

4 $$\widehat{{\overrightarrow{\underline{x}}}_{A}}=\frac{\widehat{{\overrightarrow{\underline{x}}}_{A}}}{\sqrt{tr\{{T}_{AB}^{\dagger }\,{T}_{AB}^{\dagger ,{\rm{H}}}\}}}$$

The superscripted H indicates a Hermitian transpose operation. Replacing ${\overrightarrow{\underline{x}}}_{A}$ of Eq. (1) with ${\overrightarrow{\underline{x}}}_{A}$of Eq. (3), it is possible for Bob to observe the signal directly without performing any signal processing according to the model equations:

5 $$\,\begin{array}{rcl}{\overrightarrow{y}}_{B} & = & {T}_{AB}\cdot \widehat{{\overrightarrow{\underline{x}}}_{A}}+{n}_{B}\\  & = & {T}_{AB}{T}_{AB}^{\dagger }\cdot {\overrightarrow{\underline{x}}}_{A}+{n}_{B}\\  & = & {\overrightarrow{\underline{x}}}_{A}+{n}_{B}\,\end{array}$$

**Figure 1:**
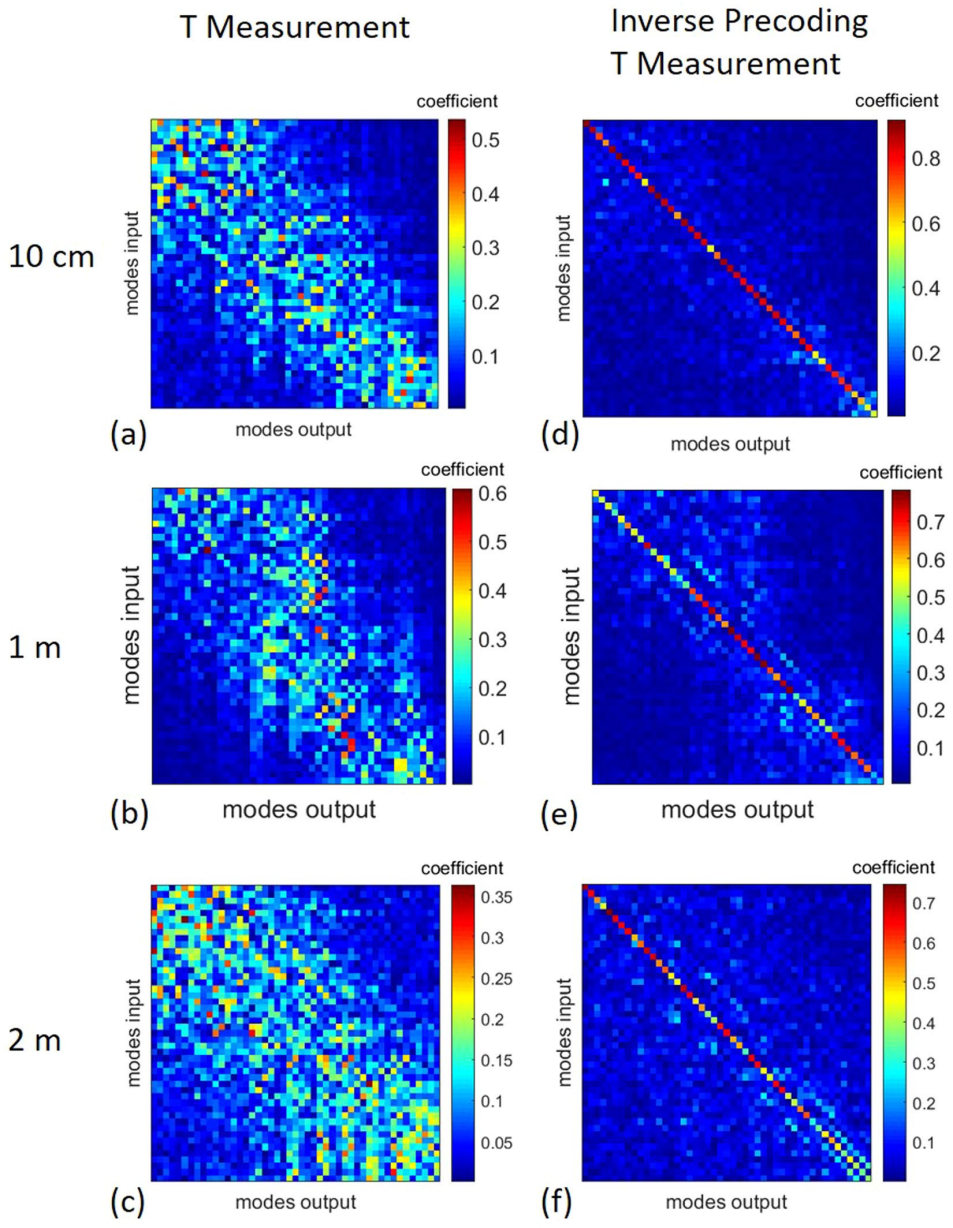
T measurements. The step-index MMFs used for the studies support 55 individual spatial modes. The mode domain of the MMFs is used as the basis for the T measurements. For this reason, the measured Ts have a dimension of 55 × 55. (**a–c**) T measurements of MMFs of different lengths, as well as (**d–f**) pre-coded diagonalized T measurements. The individual images show the pure amplitude values of the complex-valued Ts. The LPl,m modes are sorted ascending first by their l index and then by their m index: LP01, LP02, … LP11, … LPLM. (**a–c**) T measurements of 10 cm, 1 m and 2 m MMFs, respectively (**d–f**) diagonalized Ts of 10 cm, 1 m and 2 m MMFs, respectively.

Using the new input signal calculation rule [Eq. (3)], Alice gets the required combinations of complex mode weights to make a specific output signal appear on Bob’s side. In information technology this approach is known as inverse precoding^41^. Shaping such complex light field distributions requires an adaptive optical device with a high modulation depth like a spatial light modulator (SLM). In the case presented here, the SLM is utilized for simultaneous amplitude and phase modulation using superpixels^42^. If the T measurements are repeated after inverse precoding, the amplitude distribution of the T will be very similar to an identity matrix. In Fig. 1 T measurements before and after applying inverse precoding of MMFs of different lengths are shown. The efficiency of the inverse precoding process ${\eta }_{P}$ can be quantified by calculating the mean optical power, which is located on the diagonal elements of the diagonalized Ts $\overline{{P}_{d}}$ with respect to the mean optical power, which is distributed over the background entries $\overline{{P}_{b}}$^30^:

6 $${{\rm{\eta }}}_{{\rm{P}}}=\frac{\overline{{P}_{d}}}{\overline{{P}_{d}}+\overline{{P}_{b}}}$$

The efficiencies of the individual precoding processes shown in Fig. 1 are distributed as follows: the precoding of the 10 cm MMF in Fig. 1(d) has an efficiency of 95%, while the efficiency of the 1 m MMF Fig. 1(e) was 90% and 89%, respectively, for the 2 m MMF in Fig. 1(f). These results show that the inverse precoding process can also be efficiently performed on MMFs up to 2 m in length. Thus, the mean transported power in the individual channels can be transmitted with an efficiency of at least 89%.

**Figure 2:**
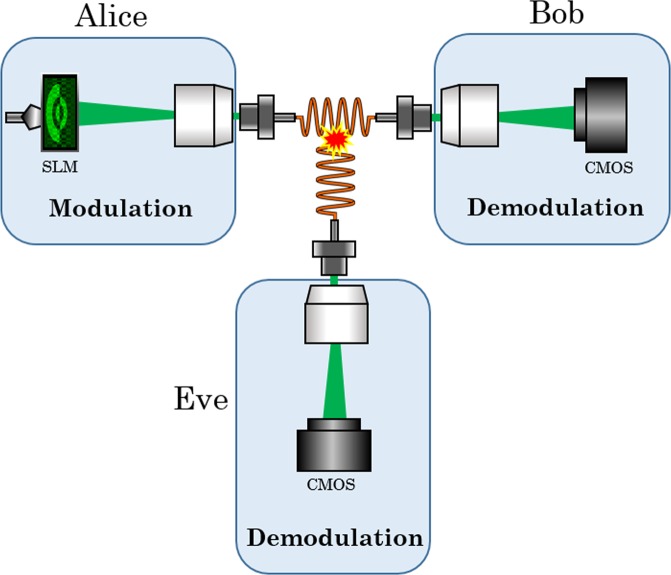
eavesdropping scenario. Eve attaches herself on the MMF used as a transmission channel between Alice and Bob. She will already listen during the calibration phase between Alice and Bob and thus knows the T between Alice and Bob. The light which is exiting on Eve’s side is of sufficient intensity, but so low that Eve’s listening process is not registered.

If Eve now gains access to the communication channel between Alice and Bob, as shown in Fig. 2, the light transmission between Alice and her is described as follows:

7 $$\,\begin{array}{rcl}{\overrightarrow{y}}_{E} & = & {T}_{AE}\cdot \widehat{{\overrightarrow{\underline{x}}}_{A}}+{n}_{E}\\  & = & {T}_{AE}{T}_{AB}^{\dagger }\cdot {\overrightarrow{\underline{x}}}_{A}+{n}_{E}\end{array}$$

whereas the noise level on Bob’s side ${n}_{B}$ has the same level as on Eve’s side ${n}_{E}$. According to^ 18,^ the probability for coupling modes into Eves tapping fiber is mode dependent. This process can be described using a diagonal matrix V, which is inserted into the transfer function, which carries the mode-dependent power loss coefficients ${\sigma }_{i}^{2}$ on the diagonal elements representing the MDL characteristic of the MMF (the determination of the exact entries of V is explained in the methods section):

8 $$V=[\begin{array}{ccc}{\sigma }_{1}^{2} & 0 & 0\\ 0 & \ddots  & 0\\ 0 & 0 & {\sigma }_{N}^{2}\end{array}]$$

where ${\sigma }_{min}^{2}$<<1 and ${\sigma }_{max}^{2}$ = 1, which results in the following transmission relation:

9 $${\overrightarrow{y}}_{E}=\sqrt{V}{T}_{AE}{T}_{AB}^{\dagger }\cdot {\overrightarrow{\underline{x}}}_{A}+{n}_{E}$$

As soon as Eve wants to decode an observed message ${\overrightarrow{y}}_{E},$ she has to invert the measured ${\rm{T}}=\sqrt{V}{T}_{AE}{T}_{AB}^{\dagger }$:

10 $$\begin{array}{rcl}\widehat{{\overrightarrow{y}}_{E}} & = & {H}^{\dagger }\cdot {\overrightarrow{y}}_{E}\\  & = & {\overrightarrow{\underline{x}}}_{A}+{H}^{\dagger }{n}_{E}.\end{array}$$

The introduced relationships lead to two important findings:

1. Due to the fact that values between 0 and 1 are located on the diagonal elements of V, the entries of H are attenuated. This will lead to noise amplification during Eve’s inversion process.
2. Due to Alice’s inverse precoding, Alice directly influences Eve’s noise term [Eq. (10)], but not Bob’s noise term [Eq. (5)].

### Security analysis of a MMF optical network using PLS

In order to examine the influence of the two findings presented in a simulation, certain assumptions are made for the introduced model using the measured Ts [Fig. 1]. First, we assume that the pure transmission characteristics are the same for both Bob and Eve. For this reason we assume for both the same measured T from Fig. 1(a) (${T}_{AE}={T}_{AB}$). In addition, it is assumed that the same additive white Gaussian noise occurs on both sides. The MDLs, which are represented by V, are the only difference between Bob’s and Eve’s model equations [Eqs. (5) and (10)]. In order to quantify the level of security using PLS, the quality of the two output signals ${\overrightarrow{y}}_{B}$ and ${\overrightarrow{y}}_{E}$ on Bob’s and Eve’s side is compared over the different mode channels with varying eavesdropping conditions. Both Bob and Eve perform thresholding during their detection, since dynamic channel changes are taken into account. While Bob can easily detect the message, due to Alice’s inverse precoding [Eq. (5)], Eve compensates for the channel by inverting the matrix she measured [Eq. (10)]. The Signal-To-Noise-Ratio (SNR) of the detected signal is given as the performance parameter:

11 $$SNR=10\cdot lo{g}_{10}(\frac{{|{\overrightarrow{y}}_{i,Signal}|}^{2}}{{|{\overrightarrow{y}}_{i,Background}|}^{2}}).$$

The inverse precoding approach gives Alice the power to manipulate Eve’s noise term. Alice thus can artificially add white Gaussian noise $\tilde{n}$ to the transmitted signal ${\overrightarrow{\underline{x}}}_{A}$ from Eq. (3):

12 $$\widehat{{\overrightarrow{\underline{x}}}_{A}}={T}_{AB}^{\dagger }\cdot ({\overrightarrow{\underline{x}}}_{A}+\tilde{n})$$

**Figure 3:**
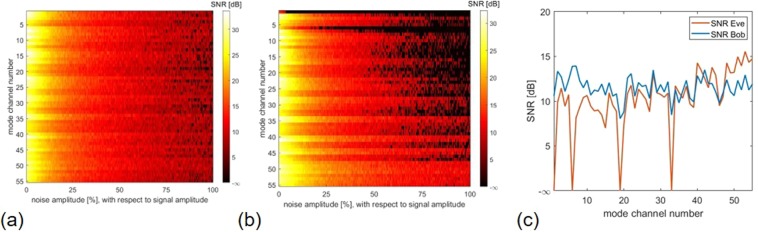
security analysis of a MMF based PLS system. The measured Ts form the basis for the simulation results. SNR for **(a)** Bob’s and **(b)** Eve’s detection respectively plotted over all 55 mode channels with increasing noise level. A ‘1’ was sent sequentially over each channels with increasing noise level and both Bob’s and Eve’s signals were evaluated. It should be noted that Eve multiplies her signal by the inverted T H^†^ before evaluation, as shown in Eq. (10). Bob, on the other hand, easily detects the signal sent by Alice due to her inverse precoding [Eq. (5)]. **(c)** line scan from a and b at a noise level of 50% at both Bob’s and Eve’s side. At this noise level, the mode channels 1, 6, 19, and 33 are safe, because Eve’s detection on these channels is faulty. However, it can also be seen that Eve has a higher SNR than Bob for the signal detection of the mode channels that are particularly favourable for her (here, for example, channels 48 and up). This is due to the fact that Eve makes an inversion at the signal detection, which is not done by Bob. It is possible that the signal powers at the channels that are favourable for Eve are amplified and ultimately higher than on Bob’s side.

It should be mentioned that both inverse precoding and adding artificial noise are linear operations. For this reason, the complexity of the implemented processes can be considered low^43^. In order to test the system for its usability, a digital ‘1’ is sent successively over each of the 55 available mode channels and the output signals on both Bob’s and Eve’s side are compared with regard to the SNR [Eq. 11]. Figure 3 shows two plots where the colour-coded SNR was calculated for both Bob’s and Eve’s sides for every available mode channel with increasing noise amplitude of $\tilde{n}$ (maximum 100% with respect to signal amplitude), respectively. As Bob and Eve use thresholding the SNR was only calculated for the case if the correct mode was detected. If the detection failed, the SNR value is set to −∞ dB artificially. If the SNR drops to −∞ dB on Eve’s side, the channel is considered safe for Alice and Bob. As can be clearly seen in Fig. 3a,b, the amplitude of the artificial noise level has a significant influence on the signal quality at Eve’s side. Bob, on the other hand, can almost consistently measure the correct signal, even if Alice adds a noise to her transmitted signal that has the same amplitude as the actual signal. If one now performs a line scan in the two SNR evaluations as shown in Fig. 3c at 50% noise, it can be seen that Bob has an almost constant SNR level of 12 dB. Additionally, there are 4 secure channels where Eve cannot decrypt the message sent by Alice at all, if Alice performs dynamic channel changes. With the presented technique the asymmetry between Bob and Eve is exploited to the highest degree, because Alice and Bob can communicate securely. By generating several secure channels it is also possible to increase the secure goodput, as it was introduced for wireless and optical communication channels in^44^. This can be achieved, for example, by adjusting the artificial noise level, since the number of secure channels can be controlled directly with it.

### Dynamic channel changes

**Figure 4:**
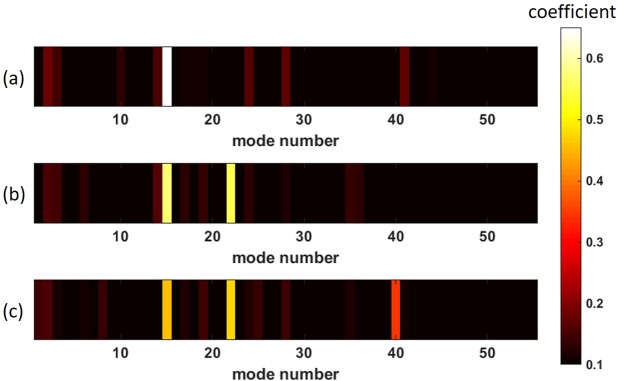
parallel MDM experiments. (**a)** one**, (b)** two and **(c)** three individual spatial fiber modes of a 1 m step-index MMF.

**Figure 5:**
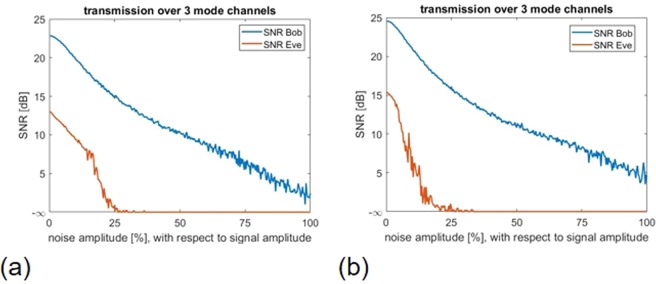
transmission over multiple channels. The measured Ts form the basis for the simulation results. SNR plotted against the increasing artificial noise level. Thresholding was used both on Bob’s and Eve’s side to detect the transmitted message (three times ‘1’) **(a)** over three safe mode channels 1, 6 and 19 and **(b)** over two safe mode channels 1, 6, as well as one unsafe mode channel 50.

The diagonalization of the MMF by means of inverse precoding not only has the advantage that Bob receives Alice’s message directly without having to perform further signal processing, but since the individual spatial fiber modes were selected as the basis of the T, MDM can be employed. Therefore, Alice is able to transmit Data on multiple arbitrary channels simultaneously. Experimental results are shown in Fig. 4. By the transmission of a light signal of constant power over several channels, the power is distributed to the individual channels. As a result, the average signal level decreases with increasing number of channels. The magnitude of the received mode coefficient in Fig. 4(a) is approximately 0.62, while the average magnitude is approximately 0.4 in Fig. 4(c). This constitutes a performance limitation of MDM in this system, which could be compensated by increasing the laser power. Nevertheless, assuming that simple thresholding is used, Alice can now choose between 55 × 54 × 53 = 157.410 transferable symbols. This corresponds to a 17 bit transmission system. Using the SLM with a repetition rate of 60 Hz, Alice could achieve a transmission rate of approximately 7.5 Mbit with a cw laser of only one wavelength. Since 3 modes can now be sent simultaneously, the effects on the two output signals of Bob and Eve are investigated for this case. The aim is to use as many channels as possible simultaneously for a particularly high data rate, but at the same time to generate a faulty detection at Eve via the inverse precoding. For this reason, in the model 3 mode channels were used simultaneously at a varying noise level and the output signals were evaluated and compared. Using 3 secure channels from Fig. 3c, it can be seen that on Eve’s side, the detection at a noise level of about 50% and above consistently fails, while Bob always receives the message in the correct way [Fig. 5a]. It is also possible to transmit a message securely, even if not all individual channels are classified as secure [Fig. 5b]. In the example shown here, it was always possible to transmit a message consisting of 3 bits securely via two secure channels and one arbitrary insecure channel. It is therefore possible to send messages with three channels from the entire mode domain as long as two channels, which are classified as safe, are involved in the message. Alice should add an artificial noise with a noise level of about 50% of the signal amplitude to her signal to ensure security.

## Discussion

The efficiency of the diagonalization via inverse precoding decreased from 95% at 10 cm to 89% at 2 m length. On the one hand, the decreasing efficiency can be explained by the fact that manufacturing tolerances play a greater role with increasing fiber lengths. The manufacturer specifies a tolerance of 10% for both the NA and the core radius. On the other hand, the efficiency of inversion changes with fiber length as the strength of the non-diagonal elements strongly increase. Thus the quality of the inverse precoding process in the investigations shown here depends on the fiber length. However, the results presented so far offer potential for short-distance applications such as data centers, however it is also possible to send data via MMFs in long-haul systems with more than 1000 km transmission distance^23^. It should be noted that the shown dependencies on fiber length can change completely if the refractive index distribution of the fiber core changes from a step-index to a gradient-index profile. Although MMFs with a step-index profile are considered to be more difficult to control than, for example, MMFs with a graded-index profile^45^ the experiments shown were nevertheless performed with a step-index MMF. This is due to the combination of the individual hardware parameters, which provide a manageable mode domain of 55 modes. This amount of modes is sufficient for proof-of-concept experiments.

A further limitation of the presented structure is the long-term stability. Since a holographic setup is used to measure the light fields, the optical system is sensitive to external perturbations such as temperature fluctuations, mechanical influences or phase drifts of the laser light used. This could lead to fading of the signal. However, it is possible to recalibrate the system with a new T measurement. This is even possible in real time, so optical fading is not considered as a problem for the presented system^46^.

The matrices measured with the introduced principle were used to perform an inverse precoding, so that communication can be done over arbitrary modes of the mode domain. Experimentally, it was shown that MDM can be performed with up to 3 independent modes via thresholding. Theoretically it would also be possible to communicate over more than 3 channels simultaneously, but this technique is limited by the inverse precoding quality. The number of SLM pixels used would be another limiting factor, but currently 130 × 130 superpixels are used for the excitation of modes, which should in principle suffice for the simultaneous excitation of all modes.

In the fundamental investigations, a basic network architecture is first chosen in which there is only one direct connection between transmitter and receiver. Future investigations will also deal with more complex architectures, but there are no reasons why the system should not be expandable.

The assumptions made for the eavesdropping scenario are favoring Eve’s role. In reality, Eve is not almighty and the coupling coefficients are not scaled down to the very optimistic value 1, i.e. completely without attenuation. Even under this hypothesis, it could be shown that if Alice adds artificial noise with 50% signal amplitude to her transmitted signal, there are 4 mode channels which can be classified as safe. Accordingly, the presented system resists an attack. The number of secure channels could, of course, change if the fiber parameters such as core radius or refractive index profile change.

## Conclusion

In this work, experimental studies on PLS in MMF optical networks are shown for the first time. PLS is a technique in which information security is not achieved by exchanging a cryptographic key, but by exploiting the physical properties of the transmission channel itself. In the example shown, PLS is implemented by using inverse precoding with artificial noise. This enables secure communication even if Eve has complete knowledge of the channels and is present during the calibration. The key for this is the exploitation of mode dependent losses which are characteristic for the channel behaviour inside MMFs. While Bob directly observes the sent modes, Eve needs to use matrix inversion. This gives Alice the power to introduce artificial noise and thus to amplify the noise Eve detects. This results in an SNR advantage for Bob, which is high enough to generate 4 mode channels in our exemplary simulation that can be considered to be secure as dynamic channel changes are used. In combination with MDM this is even enhanced. Messages can be sent safely over 3 channels from the entire mode domain, as long as 2 channels classified as safe are used. These results represent a crucial advancement for increasing the security in optical transmission channels with potential impact on data centers and cyber physical systems.

## Methods

### Optical setup used for measuring the T of a MMF

**Figure 6:**
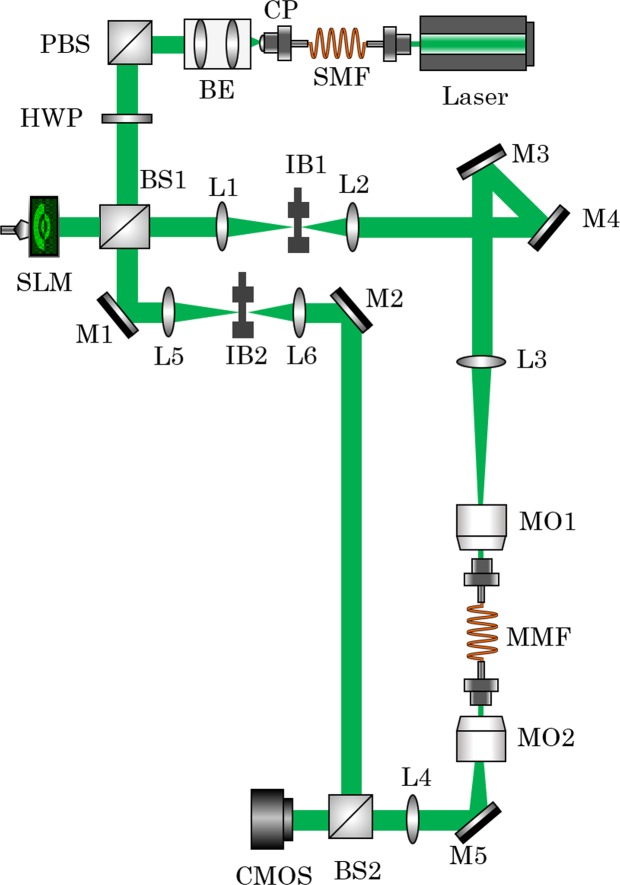
illustration of the optical setup. SMF: singlemode fiber. CP: collimation package. BE: beam expander. PBS: polarizing beam splitter. HWP: half-wave plate. SLM: spatial light modulator. BS: beam splitter. L: lens. M: mirror. IB: pinhole. MO: microscope objective. MMF: multimode fiber. CMOS: camera.

In this paper, the optical setup [Fig. 6] which has been introduced in^42^ is used to measure the T of a step-index MMF (THORLABS M68L, Ø25 µm, NA = 0.1) with exactly N measurements. The utilized light source is a 532 nm solid state laser (LaserQuantum, TORUS). This parameter combination leads to a mode domain of 55 modes per polarization direction. Mode selective excitation is performed using amplitude and phase modulation based on superpixel phase masks with a single SLM (HOLOEYE Pluto, 8 bit phase-only SLM). The light that propagates through the MMF is holographically measured using conventional off-axis digital holography. The measured complex light field is then decomposed into the MMF’s mode domain. For this step, the orthogonality of the mode field distributions in the mode domain is exploited and a complex series expansion is carried out. Using this technique, one row of the N × N sized T is measured single-shot.

### Tikhonov inversion

Tikhonov inversion is used for inverting the T, because it is robust against the influence of noise The Tikhonov inversion is performing a regularization process and has already been used in biophotonic applications^31,47^. The regularization parameter has been chosen as 12% of the maximum singular value of the T.

### Determining the loss coefficients of the coupling matrix V

**Figure 7:**
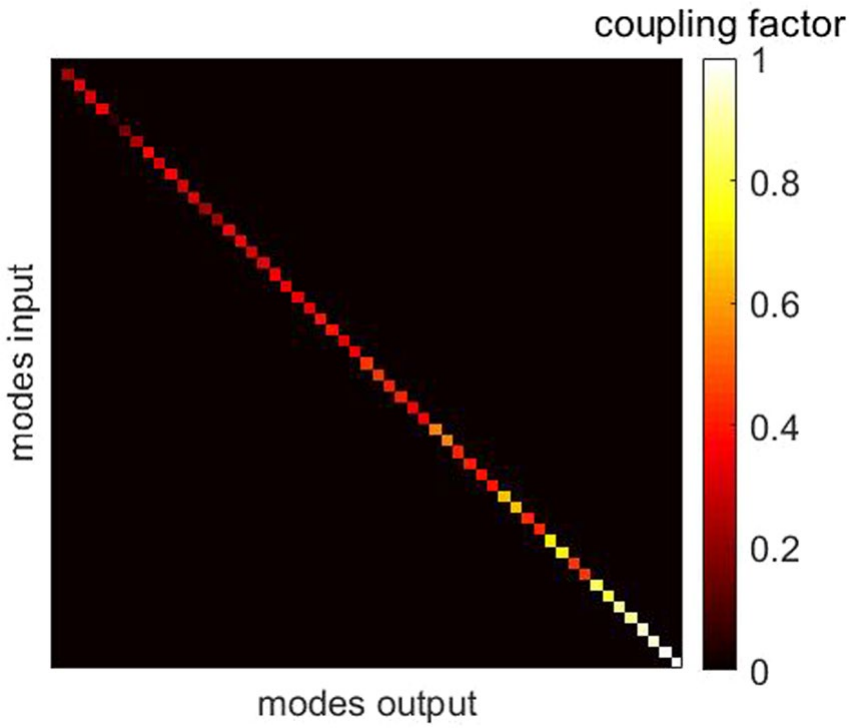
V matrix. V represents the coupling characteristic for Eve. The trend of the diagonal entries was chosen proportional to the power ratio in the edge area to the total power of the core of the respective modes.

In^18^ two different coupling matrices were considered. It was assumed that the attenuation of modes is either (i) logarithmic or (ii) linear but always randomly distributed over the individual mode channels. In order to make an accurate selection of V, power crosstalk was simulated using an Finite Difference Time Domain (FDTD) based evanescent field coupling process^48^. Individual modes of the MMF were excited in a straight MMF piece (case: transmission from Alice to Bob) and another straight MMF piece was placed next to it (case: coupling to Eve). This scenario was chosen based on the following consideration: Eve would attach the core of her MMF as close to the core of Alice’s and Bob’s MMF as she would receive sufficient intensity but would not be detected. The simulation results show that there is a deterministic relationship between the coupling behavior and the mode field distribution. The coupling process depends on the spatial distribution of the mode field power. Is the field power concentrated at the edge of the core, a particularly high proportion of the power is coupled to Eve’s fiber (6.5% in the highest order mode), whereas particularly low power is coupled for lower order modes, where the main part of the power is guided in the center of the core (0.018% in the lowest order mode). For simplicity it is now assumed that during a splicing process the highest order mode is coupled without attenuation, i.e. a coupling factor of 1 is assigned to the last entry in the V matrix. The lowest order mode experiences an attenuation of $0.018/6.5=0.0028$, which is noted in the first entry of V. The attenuation values of the modes between these extreme values were now selected according to the following scheme: the proportion of the power of the field in 10% of the edge of the core area in relation to the total total power of the core were calculated for all modes. The behaviour of this curve has now been scaled to the coupling factor values 0.0028 to 1. The result was taken as the diagonal entries of V and can be seen in Fig. 7. The same V matrix is assumed for all subsequent investigations.
